# Spatial transcriptomics identifies fibroblast–T cell crosstalk as a driver of Th2 polarization in allergic rhinitis

**DOI:** 10.3389/fimmu.2026.1788288

**Published:** 2026-04-17

**Authors:** Miao Zhao, Jiaqi Duan, Yongmin Xie, Guan Huang, Gui Yang, Pingchang Yang, Haotao Zeng

**Affiliations:** 1 Department of Clinical Chemistry, Longgang ENT Hospital, Shenzhen ENT Institute and Shenzhen Key Laboratory of ENT, Shenzhen, China; 2 Department of Otolaryngology, Longgang Central Hospital, Shenzhen, China; 3 State Key Laboratory of Respiratory Diseases, Allergy Division at Shenzhen University, Institute of Allergy and Immunology of Shenzhen University, Shenzhen Key Laboratory of Allergy and Immunology, Shenzhen, China

**Keywords:** allergy, fibroblast, nasal mucosa, spatial transcriptomics, T cell, Xenium *In Situ*

## Abstract

**Background:**

Allergic rhinitis (AR) is a common chronic nasal mucosal inflammatory disorder driven by type 2 immunity, but the spatial stromal–immune cell interactions underlying its pathogenesis remain unclear.

**Methods:**

We used 10x Genomics Xenium *In Situ* spatial transcriptomics to map the nasal mucosa of 10 AR patients and 10 non-allergic controls, combined with unsupervised cell clustering, differential gene expression (DE) analysis of COL1A1^+^PDGFRA^+^ fibroblasts, qRT-PCR validation, ligand–receptor modeling (CellPhoneDB/NicheNet), and multimodal integration of spatial, transcriptional, and clinical data.

**Results:**

Nine major cell types with tissue-specific localization were identified. The AR samples showed expanded fibroblast-rich regions (34.2 ± 3.1% vs. 15.6 ± 2.4% in controls; *p* < 0.001) and increased adjacency between CD4^+^ T cells and fibroblasts (62.3 ± 4.5% vs. 28.7 ± 3.8% in controls; *p* < 0.001). The fibroblasts in AR had 187 upregulated genes (e.g., TSLP, IL33) that were spatially enriched near CD4^+^ T cells and validated by qRT-PCR. CD4^+^ T cells within 20 μm of fibroblasts in AR showed higher Th2 cytokine expression (IL4, IL5, IL13) and Th2/GATA3 signature scores (*p* < 0.001). Three key ligand–receptor axes (TSLP–IL7R, OX40L–OX40, and ICOSL–ICOS) drove the fibroblast–Th2 crosstalk. A “fibroblast–T cell crosstalk score” was ×4.8 higher in AR (*p* < 0.001) and correlated with clinical severity (serum IgE: *r* = 0.71; SPT wheal diameter: *r* = 0.65; *p* < 0.001).

**Conclusions:**

AR is defined by expanded fibroblast niches, fibroblast-derived type 2 mediators, and ligand–receptor-dependent fibroblast–Th2 crosstalk—a central pathogenic driver and potential therapeutic target.

## Introduction

Allergic rhinitis (AR) is a highly prevalent chronic inflammatory disorder of the upper airway, affecting 20%–30% of the global population and imposing substantial burdens on quality of life, productivity, and healthcare resources (1, 2). Clinically, AR is defined by IgE-mediated hypersensitivity to environmental allergens (e.g., dust mites, pollen), manifesting as nasal congestion, rhinorrhea, sneezing, and itch (3, 4). At the immunological core, AR is driven by exaggerated type 2 helper T cell (Th2) responses: Th2 cells secrete interleukin (IL)-4, IL-5, and IL-13 (5), which orchestrate downstream pathogenic processes including B cell class switching to IgE, eosinophil recruitment, and mucus hypersecretion (5, 6). While immune cells such as dendritic cells (DCs), mast cells, and T cells are well established as contributors to AR pathogenesis (7, 8), the role of tissue-resident stromal cells—and their spatial interactions with immune cells—in shaping local Th2 polarization remains incompletely understood.

Fibroblasts, long viewed as passive structural cells that maintain tissue integrity via extracellular matrix deposition, have emerged as active regulators of immune responses across barrier tissues (skin, lung, gut) (9, 10). In allergic diseases, accumulating evidence highlights fibroblasts as key modulators of type 2 inflammation: in asthma, lung fibroblasts secrete thymic stromal lymphopoietin (TSLP) and IL-33 to prime DCs toward Th2-inducing phenotypes (6, 10). Previous studies show that fibroblasts also play an important role in allergic rhinitis (11). This study demonstrates that nasal mucosal fibroblasts secrete IL-4 to promote Th2 cell differentiation and contribute to the pathogenesis of allergic rhinitis, supported by flow cytometry sorting and RNA-sequencing analysis. We have added this key literature to strengthen the discussion of fibroblasts and immune cell interactions in allergic rhinitis, especially at the transcriptomic level (11). However, critical gaps persist in understanding fibroblast function in AR. Most studies rely on bulk RNA sequencing or single-cell RNA sequencing (scRNA-seq)—dissociative techniques that erase spatial context, making it impossible to map how fibroblasts and T cells interact *in situ* within the nasal mucosa. Whether nasal fibroblasts directly communicate with CD4^+^ T cells (rather than indirectly via DCs) to drive Th2 polarization remains untested. The specific ligand–receptor axes mediating fibroblast–T cell crosstalk in human AR nasal tissue have not been defined, and these gaps limit our ability to target stromal–immune interactions for AR therapy.

Spatial transcriptomics addresses these limitations by preserving tissue architecture while profiling gene expression, enabling the simultaneous analysis of cell type composition, spatial adjacency, and ligand–receptor communication (12, 13). 10x Genomics Xenium *In Situ*, a high-resolution targeted spatial transcriptomics platform, achieves subcellular RNA localization and single-cell resolution—uniquely suited to dissect fine-grained fibroblast–T cell interactions in AR: unlike scRNA-seq, it can resolve whether fibroblast-derived mediators colocalize with Th2-polarized T cells; unlike spot-based spatial transcriptomics, it eliminates the need for deconvolution and enables the direct quantification of cellular gene expression and spatial proximity.

In this study, we applied 10x Genomics Xenium *In Situ* to nasal mucosal biopsies from AR patients and non-allergic controls to construct a high-resolution atlas of the allergic tissue microenvironment. Our goals were to map the spatial distribution of fibroblasts and CD4^+^ T cells in AR vs. control mucosa, determine whether fibroblasts in AR upregulate type-2-skewing mediators, and identify ligand–receptor axes that mediate fibroblast–T cell crosstalk and Th2 polarization. We found that fibroblast-rich niches are significantly expanded in AR, frequently colocalize with CD4^+^ T cells, and express TSLP, OX40 ligand (OX40L), and inducible T cell costimulator ligand (ICOSL)—mediators that drive Th2 cytokine production and GATA3-dependent transcriptional programming in adjacent T cells. Collectively, our findings establish fibroblasts as critical immunomodulatory hubs in AR, positioning fibroblast–T cell crosstalk as a novel pathogenic axis and potential therapeutic target.

## Materials and methods

### Patient recruitment and sample collection

Nasal mucosa samples were collected from two cohorts of patients with nasal polyposis with or without allergic rhinitis (AR) by undergoing functional endoscopic sinus surgery at Longgang Central Hospital (Shenzhen, China). The AR group consisted of nine patients (five male and four female; age range: 22–45 years; mean ± SEM: 31.2 ± 2.5 years) with physician-diagnosed AR and nasal polyps. A diagnosis of AR was confirmed by the presence of clinical symptoms (nasal congestion, rhinorrhea, sneezing, itch) for ≥6 months, positive skin prick test (SPT) to ≥1 common aeroallergen (dust mites: *Dermatophagoides pteronyssinus*, *Dermatophagoides farinae*; pollen: *Artemisia*, ragweed; pet dander: cat, dog), and serum total IgE >0.35 IU/mL (measured via chemiluminescent immunoassay, Beckman Coulter DXI 800). Mean AR duration (time since diagnosis) was 4.8 ± 0.7 years. The control group included nine patients (five male and four female; age range: 20–48 years; mean ± SEM: 33.5 ± 3.1 years) with no history of allergic or autoimmune disease, negative SPT to all tested allergens, and serum total IgE <0.35 IU/mL and who underwent surgery for the removal of nasal polyposis.

The exclusion criteria for all participants included the use of systemic corticosteroids within 4 weeks of biopsy, topical nasal steroid use within 2 weeks of biopsy, active upper respiratory tract infection (e.g., sinusitis) at enrollment, comorbidities including asthma, chronic obstructive pulmonary disease, or immunodeficiency, and prior nasal surgery within 6 months.

Nasal mucosal samples (3–5 mm in diameter) were obtained from surgically removed nasal tissues. The samples were immediately placed in ice-cold RPMI-1640 medium (Thermo Fisher Scientific, catalog no. 11875093) supplemented with 1% penicillin–streptomycin (Thermo Fisher, catalog no. 15140122) and transported to the laboratory within 30 min. Upon arrival, the samples were embedded in optimal cutting temperature (OCT) compound (Sakura Finetek, catalog no. 4583), snap-frozen in liquid nitrogen, and stored at –80 °C until cryosectioning.

All patients provided written informed consent. The study was approved by the Institutional Review Board of Longgang Central Hospital (Protocol No. 2025ECPJ042), and conducted in accordance with the Declaration of Helsinki.

### Tissue sectioning and histology

Frozen OCT-embedded tissue blocks were equilibrated to –20°C in a Leica CM3050S cryostat (Leica Biosystems) for 10 min before sectioning. Serial 10-μm-thick sections were mounted onto 10x Genomics Xenium *In Situ* Slides (catalog no. 1000466) and immediately stored at –80 °C to prevent RNA degradation. For morphological assessment, adjacent 10-μm sections were mounted onto glass slides, fixed in 4% paraformaldehyde (Sigma-Aldrich, catalog no. P6148) for 10 min at room temperature (RT), and stained with hematoxylin (Sigma-Aldrich, catalog no. H3136) for 5 min and eosin (Sigma-Aldrich, catalog no. E4009) for 30 s. The stained sections were dehydrated via a graded ethanol series (70%, 95%, and 100%; 2 min each) and cleared in xylene (Sigma-Aldrich, catalog no. X5800) for 5 min. The slides were mounted with Permount mounting medium (Thermo Fisher, catalog no. SP15-100) and imaged on a Leica Aperio AT2 digital slide scanner at ×40 magnification (0.25 μm/pixel resolution).

### Nasal mucosa paraffin section and H&E staining procedures

#### Nasal mucosa tissue processing and paraffin sectioning

Nasal mucosal tissue samples were immediately fixed in 4% paraformaldehyde (PFA) at 4 °C for 24–48 h, followed by dehydration through a graded ethanol series (70% → 80% → 95% → 100% ethanol), clearing in xylene, and infiltration with molten paraffin wax. The embedded tissue blocks were sectioned at 4- to 5-μm thickness using a rotary microtome, and the sections were mounted on positively charged glass slides and then dried at 60 °C for 1 to 2 h to ensure adhesion.

#### Hematoxylin and eosin staining

Mounted paraffin sections were deparaffinized in xylene (2 × 10 min) and rehydrated through graded ethanol (100% → 70% → distilled water). The sections were stained with hematoxylin for 3–5 min to label cell nuclei, followed by differentiation in 1% acid alcohol and bluing in Scott’s tap water substitute. Subsequently, the sections were counterstained with eosin for 1–3 min to stain the cytoplasm and extracellular matrix. After dehydration in graded ethanol and clearing in xylene, the slides were mounted with a coverslip using a neutral mounting medium for microscopic examination.

### Xenium *In Situ* spatial transcriptomics assay

The Xenium *In Situ* assay was performed using the 10x Genomics Xenium *In Situ* Reagent Kit (Human) (catalog no. 1000463) and a custom-designed gene panel (targeting 412 genes, including canonical cell type markers, type 2 immunity-related cytokines, costimulatory molecules, and ECM proteins) following the manufacturer’s standard protocol (CG000714, Rev A). The key steps were as follows:

1. Tissue preparation: Xenium slides with frozen sections were fixed in cold 4% paraformaldehyde (RT, 10 min), permeabilized with proteinase K (10x Genomics, catalog no. 1000464) at 37 °C for 12 min (optimized for nasal mucosal tissue), and post-fixed in 4% paraformaldehyde (RT, 5 min).
2. Probe hybridization: Target-specific Xenium oligo probes were hybridized to tissue sections (37 °C, 16 h) to enable specific RNA capture and barcoding.
3. Rolling circle amplification (RCA): Captured RNA was amplified via RCA (37 °C, 2 h) to generate DNA amplicons for fluorescent detection.
4. Cyclic imaging and decoding: Fluorescent imaging and barcode decoding were performed on the 10x Genomics Xenium Analyzer (v1.0) with ×40 magnification, achieving 0.2-μm subcellular RNA localization precision and single-cell resolution.
5. Onboard cell segmentation: Multimodal cell segmentation (integrating DAPI nuclear staining, membrane marker staining, and RNA transcript spatial coordinates) was performed using Xenium Onboard Analysis (v1.4) to define single-cell boundaries and quantify transcript counts per cell.

### Data processing and quality control

Raw Xenium imaging and transcript data were processed using the 10x Genomics Xenium Explorer (v1.4) and Xenium Pipeline (v1.4) with the GRCh38 human reference genome (GENCODE v38). The key quality control (QC) steps included the following:

1. Transcript-level filtering: Removal of low-quality transcripts (signal intensity <200 AU, misaligned barcodes).
2. Cell-level filtering: Exclusion of cells with <50 detected genes, >20% mitochondrial gene content (to exclude necrotic/apoptotic cells), or <100 total transcripts (to exclude empty segmentation masks).

After filtering, 3,500–6,000 single cells were retained per sample (mean: 4,800 cells/sample), with an average of 125 ± 30 genes and 850 ± 150 transcripts per cell.

Filtered single-cell gene expression matrices (transcript counts per gene per cell) were imported into Seurat (v4.3.0, R v4.5.1). Data were log-normalized using ‘NormalizeData’ (scale factor = 10,000), and variable features were identified using ‘FindVariableFeatures’ (selection.method = “vst”, nfeatures = 2,000). To correct for batch effects across samples, data were integrated using Seurat’s ‘IntegrateData’ function (dims = 1:30, k.filter = 200) with canonical correlation analysis (CCA).

### Cell type annotation

Integrated data were scaled using ‘ScaleData’ (vars.to.regress = “percent.mt” to correct for mitochondrial contamination), followed by principal component analysis (PCA) using the top 2,000 variable features. The first 30 principal components (PCs) were retained for downstream analysis (determined via `ElbowPlot`). Uniform manifold approximation and projection (UMAP) was used for visualization (dims = 1:30, n.neighbors = 30, min.dist = 0.3).

Cell clusters were identified using the Louvain algorithm (‘FindClusters’, resolution = 0.6), resulting in 12–15 clusters per sample. The clusters were assigned to cell types based on the average expression of canonical marker genes (log2 fold change >1.5 vs. other clusters, adjusted *p* < 0.05) and spatial overlap with histological features. The marker genes for each cell type were as follows:

- Epithelial cells: EPCAM, KRT18, MUC5AC
- Fibroblasts: Fibroblasts were annotated using the canonical pan-fibroblast markers COL1A1 and PDGFRA (co-expressed with an additional fibroblast marker POSTN), which are universally used for fibroblast identification in human respiratory mucosal tissue and exhibit no significant expression in other cell types. These markers were used to define the total fibroblast pool (not phenotypic subtypes) in nasal mucosa samples.
- Endothelial cells: PECAM1, VWF, CDH5
- Macrophages: CD68, CD163, MRC1
- Dendritic cells (DCs): LYZ, CD1C, CLEC9A
- Mast cells: TPSAB1, CMA1, KIT
- B cells: MS4A1, CD79A, IGHD
- CD4^+^ T cells: CD3E, CD4, FOXP3 (for Tregs)
- CD8^+^ T cells: CD3E, CD8A, GZMB

### Differential gene expression and spatial statistics

Differential expression (DE) analysis between AR and control fibroblasts was identified using Seurat’s ‘FindMarkers’ function (test.use = “wilcox”, logfc.threshold = 0.25, min.pct = 0.1). The Benjamini–Hochberg method was used to correct for false discovery rate (FDR); genes with adjusted *p <*0.05 and log2 fold change >0.25 were considered significant.

With reference to published studies (14), spatial adjacency between fibroblasts and CD4^+^ T cells was quantified using the Squidpy Python library (v1.2.2) with single-cell spatial coordinates (Xenium output). A “neighborhood” was defined as cells within a Euclidean distance of 20 μm (physiologically relevant for paracrine cell–cell communication). Proximity scores were calculated via permutation testing (1,000 iterations, null model: random permutation of cell type labels across spatial coordinates); *p <*0.01 was considered significant.

A distance threshold of 20 µm was used to define CD4^+^ T cell–fibroblast adjacency. This threshold was selected based on established biological evidence indicating that key soluble mediators of allergic inflammation (TSLP, IL-33, CCL17) and juxtacrine co-stimulatory signals (OX40-OX40L, ICOS-ICOSL) operate within a 10–20-µm radius in mucosal tissue microenvironments (14). This biologically informed cutoff ensures the identification of cells within a functionally relevant signaling range, reflecting potential paracrine or physical crosstalk.

The density of CD4^+^ T cells expressing IL4, IL5, or IL13 was quantified in fibroblast-adjacent (≤20 μm) vs. fibroblast-poor (>20 μm) regions. Differences were tested using the Wilcoxon rank-sum test (*p* < 0.05).

Proximity scores quantifying the spatial adjacency between fibroblasts and CD4^+^ T cells were calculated using the squidpy.gr.spatial_neighbors and squidpy.gr.nhood_enrichment functions (Squidpy v1.2.2), with a biologically informed 20-μm Euclidean distance threshold defining cellular neighborhoods (as justified in the revised “Materials and methods”). The permutation test workflow for proximity score and *p*-value generation was performed as follows:

Observed enrichment score calculation: For each sample, we first quantified the observed number of fibroblast-CD4^+^ T cell neighborhood pairs (i.e., CD4^+^ T cells within 20 μm of fibroblasts) and normalized this count to the total number of cellular neighborhood pairs in the tissue to generate an observed enrichment score (a value >1 indicates significant colocalization of the two cell types).

Null distribution generation: We performed 1,000 iterative permutations of cell type labels across all single-cell spatial coordinates in the tissue (preserving the original spatial density and cellular distribution, only randomizing cell type identities). For each permutation, we recalculated the enrichment score to generate a null distribution of enrichment scores reflecting the expected spatial association of fibroblasts and CD4^+^ T cells by chance alone.

Proximity score and raw *p*-value derivation: The final proximity score for each sample was defined as the ratio of the observed enrichment score to the mean of the null enrichment distribution (a higher ratio indicates stronger non-random spatial adjacency). A raw one-tailed *p*-value was calculated by determining the proportion of permuted null enrichment scores that were greater than or equal to the observed enrichment score (testing the alternative hypothesis of positive colocalization).

### Multiple testing adjustment for permutation-based *P*-values

To account for multiple testing across all cell type pairs (fibroblasts vs. CD4^+^ T cells, CD8^+^ T cells, macrophages, dendritic cells, mast cells, B cells, epithelial cells, and endothelial cells) analyzed in our spatial adjacency assays, we applied the Benjamini–Hochberg (BH) false discovery rate (FDR) correction to the raw permutation-based *p*-values (FDR-adjusted *p <*0.01 was set as the significance threshold). This adjustment controls for the expected proportion of false positive results across all cell type pair comparisons and ensures the statistical rigor of our fibroblast-CD4^+^ T cell adjacency findings.

### Gene module and Th2 signature scoring

Th2 transcriptional signatures were quantified using Seurat’s ‘AddModuleScore’ function. Two gene sets were used, namely:

1. A core Th2 cytokine set including IL4, IL5, IL13, IL9 [curated from prior AR studies (5, 6)]
2. A GATA3 target set consisting of 42 genes with validated GATA3 binding sites [from MSigDB Hallmark “TH2_CELL_DIFFERENTIATION” [M5938] and ChIP-seq data (15)]

The module scores were normalized to a range of 0–1. Differences in scores between fibroblast-associated and fibroblast-poor T cells were tested using a two-sided Student’s *t*-test (*p* < 0.05).

### Ligand–receptor interaction analysis

Putative fibroblast–CD4^+^ T cell interactions were inferred using CellPhoneDB (v3.0.0, database v4.1), a tool that prioritizes interactions based on the co-expression of ligands (fibroblasts) and receptors (CD4^+^ T cells). Input cell groups were defined as fibroblasts and CD3E^+^CD4^+^ T cells (single-cell populations from Xenium data). Significant interactions were identified via permutation testing (10,000 iterations, FDR-adjusted *p* < 0.05).

NicheNet (v2.0.0) was used to predict downstream target genes in CD4^+^ T cells activated by fibroblast-derived ligands. Ligands with CellPhoneDB-adjusted *p* < 0.01 were used as input; target genes were prioritized based on ligand–target gene regulatory potential (top 5% of scores). Enrichment of Th2-associated targets was tested using Fisher’s exact test (adjusted *p* < 0.05).

### Fibroblast ligand score and crosstalk score calculation

To quantify the functional impact of fibroblast–CD4^+^ T cell spatial proximity, we derived two complementary metrics: a single-cell fibroblast ligand score (for mechanistic pairing) and a sample-level fibroblast–T cell crosstalk score (for clinical correlation).

### Single-cell fibroblast ligand score

(1) For each CD4^+^ T cell in the Xenium *In Situ* dataset, we identified its nearest fibroblast neighbor within a 20-μm Euclidean distance threshold (defined as physiologically relevant for paracrine signaling, as justified in the “Spatial statistics” subsection). The single-cell fibroblast ligand score for each CD4^+^ T cell was calculated as the sum of normalized expression values of five key pro-Th2 fibroblast ligands (TSLP, ICOSL, OX40L, CCL17, and POSTN) in this paired nearest fibroblast:


$$\begin{array}{l} \mathrm{Fibroblast}\;\mathrm{ligand}\;\mathrm{score}\;=\;\sum i\\ \in {\left\{TSLP,ICOSL,OX40L,CCL17,POSTN\right\}}^{\;normExpr}i \end{array}$$

This score represents the pro-Th2 signaling potential of the fibroblast immediately adjacent to a given CD4^+^ T cell and was used for single-cell correlation analyses with T cell GATA3 and IL-4 expression.

**Figure 1:**
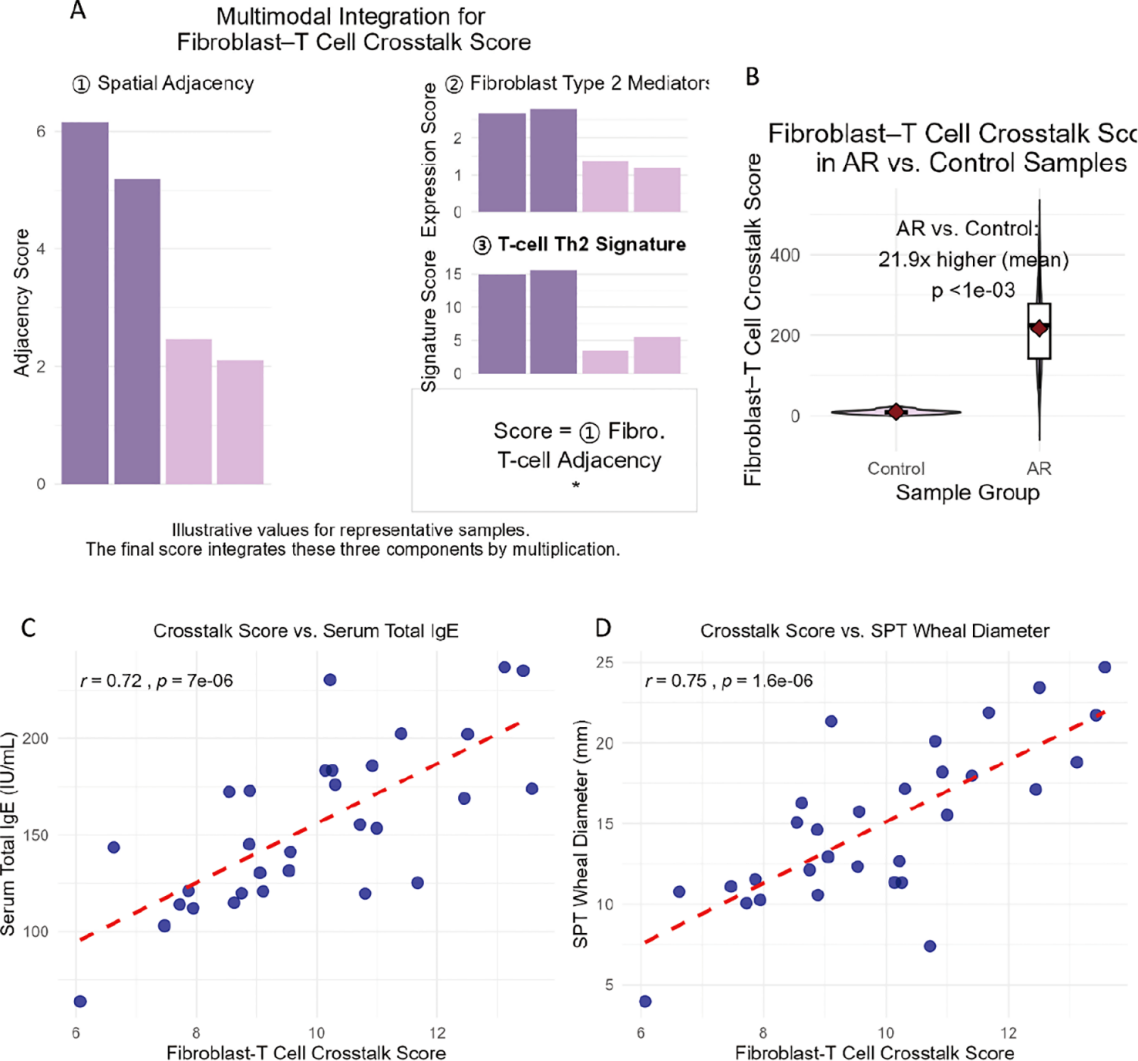
Multimodal integration of spatial, transcriptomic, and clinical data reveals fibroblast–T cell crosstalk as a driver of AR pathogenesis. **(A)** Components of the fibroblast–T cell crosstalk score. Illustrative bar graphs demonstrate the three integrated components contributing to the overall crosstalk score: (1) spatial adjacency (representing proximity of fibroblasts to CD4^+^ T cells), (2) fibroblast type 2 mediator expression score, and (3) T-cell Th2 signature score. The final composite score for each sample is calculated as the product of these three components. **(B)** Elevated fibroblast–T cell crosstalk score in allergic rhinitis (AR) samples. Violin–boxplot showing the distribution of the fibroblast–T cell crosstalk score in control versus AR patient samples. The mean crosstalk score was 21.9-fold higher in AR samples compared to control samples (*p* < 1e-03, unpaired *t*-test). Individual data points are shown within the violins, with red diamonds indicating the mean and its 95% confidence interval. **(C, D)** Sample-level correlation between fibroblast–T cell crosstalk score (X-axis, sample-level average of single-cell fibroblast ligand scores) and clinical severity metrics (Y-axis) in AR patients (*n* = 30). Each data point represents an individual patient. **(C)** Serum total IgE levels (IU/mL). **(D)** Skin prick test (SPT) wheal diameter (mm) to aeroallergens. Pearson correlation coefficients and *p*-values are shown.

(2) Sample-level fibroblast–T cell crosstalk score: To enable correlation with clinical severity metrics, we aggregated single-cell ligand scores at the patient sample level. The sample-level crosstalk score for each AR patient was defined as the arithmetic mean of all single-cell fibroblast ligand scores across all CD4^+^ T cell–fibroblast pairs (within 20 μm) in that patient’s nasal mucosal sample:


$$\mathrm{Crosstalk}\;\mathrm{score}\;=\;N1\;\sum j=1N^{Fibroblast\;ligand\;score}j$$

where *N* is the total number of CD4^+^ T cell–fibroblast pairs in the sample. This sample-level score reflects the average pro-Th2 signaling potential of fibroblast–T cell interactions in the nasal mucosa of each patient and was used for correlation analyses with serum total IgE levels and skin prick test (SPT) wheal diameters (**Figures 1C, D**).

All score calculations were performed using custom R scripts (v4.2.1) and the Squidpy Python library (v1.2.2) for spatial neighbor identification, with normalized gene expression values derived from the Xenium *In Situ* platform’s built-in UMI counting and normalization pipeline.

### Data processing and quality control

Raw Xenium imaging and transcript data were processed using the 10x Genomics Xenium Explorer (v1.4) and Xenium Pipeline (v4.1) with the GRCh38 human reference genome (GENCODE v38). Stringent, multi-level quality control (QC) was performed at the raw image, transcript, and single-cell levels to eliminate technical noise and ensure data reliability, with all filtering parameters pre-defined and applied uniformly across all AR and control samples (10 per group) to avoid batch effects. The key QC steps were as discussed below.

### Raw imaging data QC

Prior to transcript calling, Xenium raw imaging data (fluorescent signal stacks) were quality-checked for imaging artifacts (e.g., tissue folding, non-specific fluorescence, signal saturation) and tissue integrity using Xenium Explorer. Images with >10% tissue folding or signal saturation in the central mucosal region were excluded. Background fluorescence was subtracted using the Xenium Pipeline’s built-in rolling-ball algorithm (radius = 5 pixels) to normalize signal intensity across tissue sections.

### Transcript-level filtering

After initial transcript calling by the Xenium Pipeline, low-quality transcripts were rigorously filtered out using two criteria: (1) fluorescent signal intensity <200 arbitrary units (AU, a threshold optimized for nasal mucosal tissue based on 10x Genomics technical guidelines and our pre-experiment validation) and (2) misaligned barcodes (barcode sequences with >2 mismatches to the reference probe library). Only high-confidence, uniquely mapped transcripts were retained for downstream analysis.

### Cell-level filtering

Single-cell segmentation masks generated by using Xenium Onboard Analysis (v1.4) were further filtered to exclude non-viable cells, empty segmentation, and low-quality cellular profiles, with three strict exclusion criteria, namely:

1. <50 detected genes per cell (to exclude fragmented or low-RNA-content cells)
2. 20% mitochondrial gene content (to exclude necrotic/apoptotic cells, using MT- gene family as mitochondrial markers)
3. <100 total transcripts per cell (to exclude empty segmentation masks and non-cellular signal clusters)

### Sample-level QC

At the sample level, we excluded any sample with <3,000 retained single cells after transcript and cell-level filtering (to ensure sufficient cellular depth for unsupervised clustering and differential expression analysis). No samples were excluded based on this criterion in the present study.

After complete multi-level QC, 3,500–6,000 single cells were retained per sample (mean: 4,800 cells/sample), with an average of 125 ± 30 genes and 850 ± 150 transcripts per cell across all samples. These QC metrics are consistent with 10x Genomics’ recommended quality standards for Xenium *In Situ* assays in human mucosal tissues, confirming the high quality and reliability of our spatial transcriptomic data.

Filtered single-cell gene expression matrices (transcript counts per gene per cell) were imported into Seurat (v4.3.0, R v4.5.1). Data were log-normalized using NormalizeData (scale factor = 10,000), and variable features were identified using FindVariableFeatures (selection.method = “vst”, nfeatures = 2,000). To correct for batch effects across samples, data were integrated using Seurat’s IntegrateData function (dims = 1:30, k.filter = 200) with canonical correlation analysis (CCA).

### Statistical analysis

All statistical tests were performed in R (v4.5.1). Data are presented as mean ± SD unless otherwise stated. For two-group comparisons, parametric data (e.g., age, module scores) were analyzed using unpaired Student’s *t*-test, and nonparametric data (e.g., transcript counts, proximity scores) were analyzed using Wilcoxon rank-sum test. For multiple comparisons, one-way ANOVA with Tukey’s *post hoc* test was used. A *p*-value <0.05 was considered statistically significant for all analyses, except for permutation-based tests (CellPhoneDB, spatial proximity) where FDR-adjusted *p <*0.05 was used.

## Results

### Spatial atlas of the nasal mucosa reveals expanded fibroblast–T cell niches in AR

Representative H&E-stained sections of nasal mucosa from allergic rhinitis (AR) patients revealed characteristic pathological alterations of chronic type 2 inflammation. The superficial pseudostratified columnar epithelium remained largely intact but exhibited mild hyperplasia and nuclear crowding, consistent with persistent inflammatory stimulation. The underlying lamina propria was markedly expanded by dense infiltration of mixed immune cells, including lymphocytes, plasma cells, and scattered eosinophils—key effector cells of type 2 allergic immunity. Concomitantly, stromal edema and vascular dilation were evident, reflecting increased vascular permeability and tissue remodeling driven by inflammatory mediator release. These histological features collectively recapitulate the core structural and immunopathological changes of allergic nasal mucosa, aligning with the spatial transcriptomic and immune cell profiling data presented herein (**Supplementary Figure S1**).

**Figure 2:**
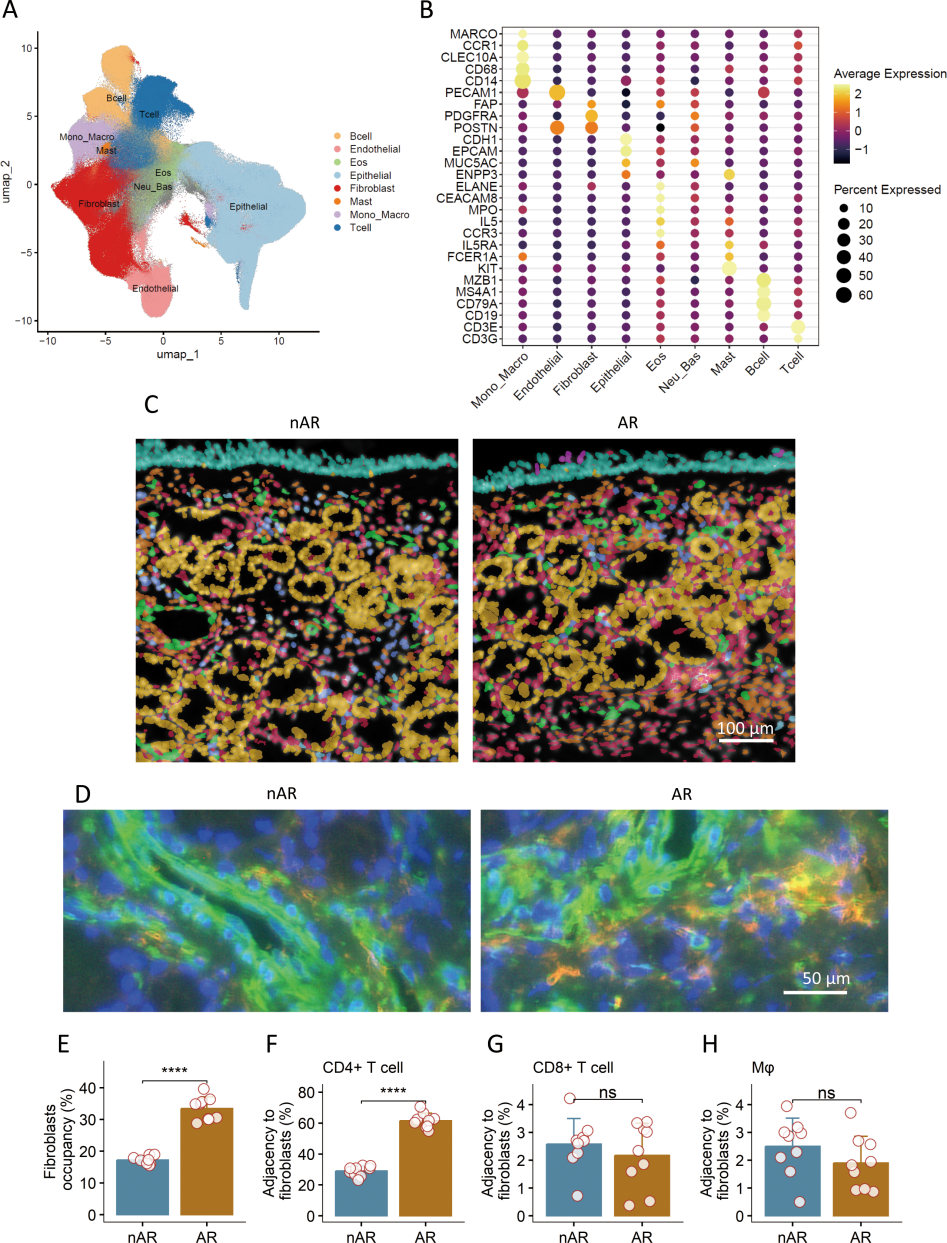
Spatial cell atlas of the nasal mucosa in allergic rhinitis (AR). This figure presents a comprehensive spatial cell atlas of the nasal mucosa, defining key cell populations and their spatial relationships in allergic rhinitis (AR). **(A)** Unsupervised clustering of spatial transcriptomic data identified major structural and immune cell populations, including epithelial cells, fibroblasts, endothelial cells, and multiple immune cell subsets. Uniform manifold approximation and projection (UMAP) plots visualize these annotated cell populations across AR and non-allergic rhinitis (nAR) samples. **(B)** Bubble plots display the average expression levels of canonical marker genes, validating the identity of each cell population within the nasal mucosa. **(C)** Spatial maps illustrate the distinct localization of immune cells in nasal tissues and the intricate fibroblast networks within the underlying stromal compartment. **(D)** Immunohistochemical images demonstrate the aggregation of CD4^+^ T cells (red) around fibroblasts (green) in the nasal mucosa of patients with allergic rhinitis (AR). **(E)** Quantification of fibroblast occupancy in the nasal mucosa. **(F–H)** Analysis of the adjacency of CD4^+^ T cells **(F)**, CD8^+^ T cells **(G)**, and macrophages **(H)** to fibroblasts in the nasal mucosa. Data in bar graphs are presented as mean ± standard deviation (SD). Each dot represents an individual sample. Statistical analysis was performed using Student’s *t*-test. ****p < 0.0001; ns, not significant.

To establish a single-cell resolution spatially resolved reference of the allergic nasal mucosa, we generated Xenium *In Situ* spatial transcriptomic profiles from 10 AR patients and 10 non-allergic controls. Unsupervised clustering and marker-gene-based annotation identified nine major cell types, consistent across all samples: epithelial cells (EPCAM^+^, KRT18^+^), fibroblasts, endothelial cells (PECAM1^+^, VWF^+^), macrophages (CD68^+^, CD163^+^), dendritic cells (DCs; LYZ^+^, CD1C^+^), mast cells (TPSAB1^+^, CMA1^+^), B cells (MS4A1^+^, CD79A^+^), CD4^+^ T cells (CD3E^+^, CD4^+^), and CD8^+^ T cells (CD3E^+^, CD8A^+^) (**Figures 2A, B**).

Spatial mapping (single-cell resolution) confirmed the tissue-specific localization of these cell types: epithelial cells were restricted to the mucosal surface (**Figure 2C**, top), while fibroblasts and endothelial cells formed a dense stromal network beneath the epithelium (**Figure 2C**, middle). Immune cells—including CD4^+^ T cells and macrophages—were distributed across both epithelial and stromal layers but showed preferential colocalization with fibroblasts in AR samples (**Figures 2C, D**).

Quantitative analysis revealed two key differences between AR and control mucosa. Fibroblast-rich regions were significantly expanded in AR, as fibroblasts occupied 34.2 ± 3.1% of total tissue area in AR samples compared to 15.6 ± 2.4% in controls (*p* < 0.001, unpaired *t*-test; **Figure 2E**). CD4^+^ T cells showed enhanced adjacency to fibroblasts in AR: using a 20-μm proximity threshold (physiologically relevant for paracrine signaling), we found that 62.3 ± 4.5% of CD4^+^ T cells were adjacent to fibroblasts in AR versus 28.7 ± 3.8% in controls (*p* < 0.001, permutation test; **Figure 2F**). This adjacency was not observed for other immune cells (e.g., CD8^+^ T cells, macrophages), indicating a specific fibroblast–CD4^+^ T cell interaction in AR (**Figures 2G, H**).

These data provide the first single-cell spatial resolution evidence that AR is characterized by expanded fibroblast niches and preferential fibroblast–CD4^+^ T cell colocalization—establishing a structural framework for stromal-mediated immune polarization.

### Fibroblasts in AR upregulate type 2-skewing mediators

**Figure 3:**
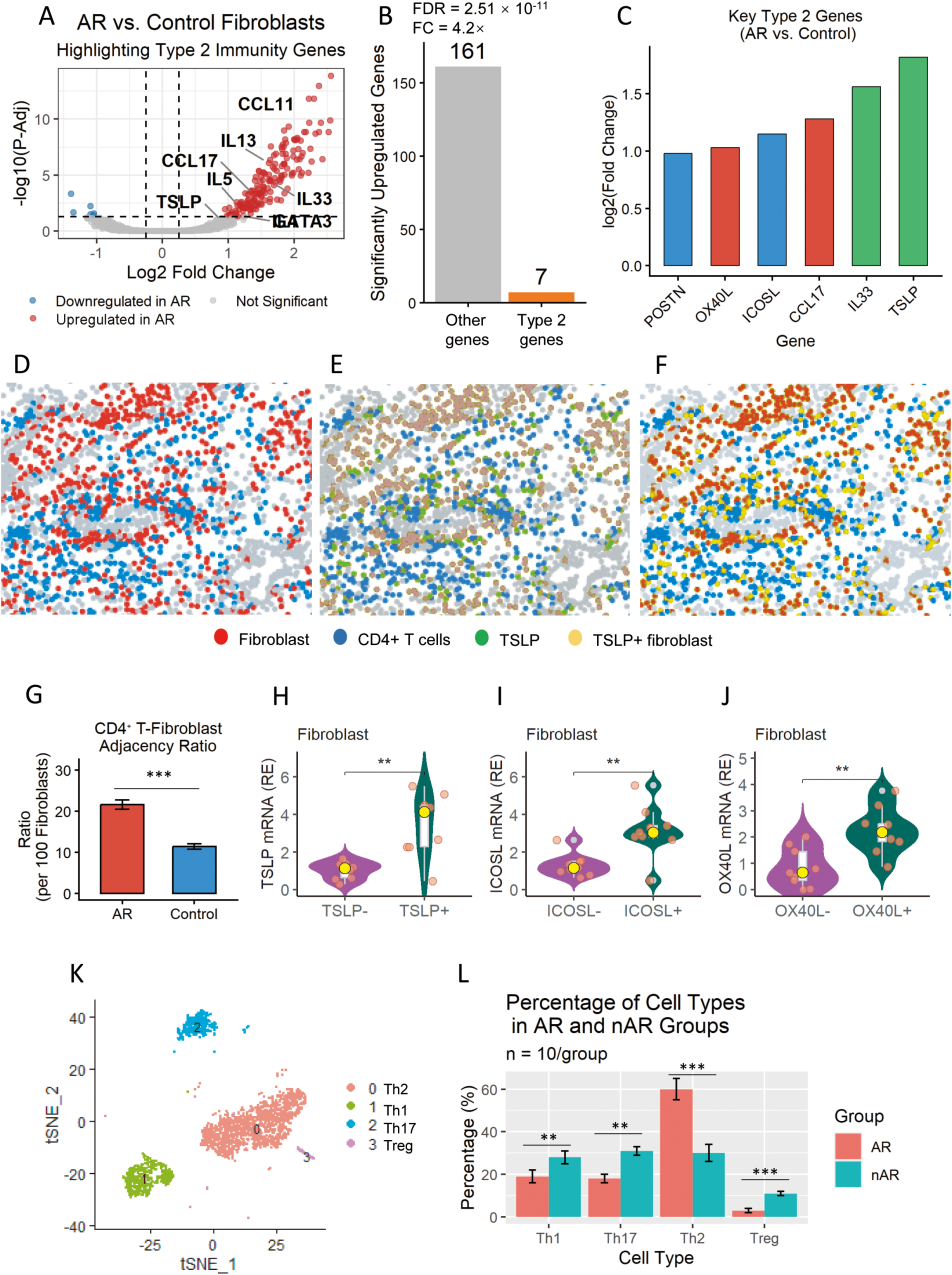
Type-2 immunity signature is selectively upregulated in airway fibroblasts of allergic rhinitis (AR) patients. **(A)** Volcano plot of differential expression in fibroblasts (AR vs. healthy controls). Dots represent genes; x-axis = log_2_ fold-change (log_2_FC), y-axis = –log_10_ adjusted *P*-value. Horizontal dashed line: adj. *P* = 0.05; vertical lines: |log_2_FC| = 0.25. Significantly upregulated genes (adj. *P* < 0.05, log_2_FC > 0.25) are red; key type-2–associated transcripts are labeled. **(B)** Breakdown of the 187 upregulated genes. Genes with annotated type-2 immune function; gray bars: remaining upregulated genes, showing the prominent type-2 enrichment in AR fibroblasts. Pathway enrichment analysis was performed with a hypergeometric test (BH-FDR correction) and reported the exact statistical values for Th2 pathway enrichment (fold change = ×4.2, FDR = 2.51 × 10^−11^). **(C)** Log_2_ fold change of top pro-Th2 genes in AR pan-fibroblasts. **(D–F)** Spatial proximity of TSLP-producing fibroblasts and CD4^+^ T cells in AR nasal mucosa by multiplex imaging. **(D)** Fibroblasts **(red)** and CD4^+^ T cells (blue). **(E)** CD4^+^ T cells (blue) and TSLP protein (green). **(F)** Overlay: fibroblasts (red), CD4^+^ T cells (blue), and TSLP^+^ fibroblasts (yellow; merged red + green). **(G)** Bar plot showing the normalized CD4^+^ T cell–fibroblast adjacency ratio, defined as the number of CD4^+^ T cells within 20 μm of each fibroblast (per 100 fibroblasts) in nasal mucosal tissue from allergic rhinitis (AR) patients and non-allergic controls. **(H)** Violin plot showing the relative expression (RE) of *TSLP* mRNA in fibroblasts from TSLP-negative (TSLP−, purple) and TSLP-positive (TSLP+, dark green) samples. The yellow dot represents the median, and individual data points are overlaid. A significant increase in *TSLP* expression was observed in TSLP+ fibroblasts compared to TSLP− fibroblasts (**p < 0.01). **(I)** Violin plot showing the relative expression (RE) of *ICOSL* mRNA in fibroblasts from ICOSL-negative (ICOSL−, purple) and ICOSL-positive (ICOSL+, dark green) samples. The yellow dot represents the median, and individual data points are overlaid. *ICOSL* expression was significantly higher in ICOSL+ fibroblasts compared to ICOSL− fibroblasts (**p < 0.01). **(J)** Violin plot showing the relative expression (RE) of *OX40L* mRNA in fibroblasts from OX40L-negative (OX40L−, purple) and OX40L-positive (OX40L+, dark green) samples. The yellow dot represents the median, and individual data points are overlaid. *OX40L* expression was significantly elevated in OX40L+ fibroblasts compared to OX40L− fibroblasts (**p < 0.01). **(K)** t-SNE visualization of CD4^+^ T helper cell subsets from peripheral blood mononuclear cells (PBMCs) of AR and nAR patients. The clusters are color-coded: Th2 (red), Th1 (green), Th17 (cyan), and Treg (purple). The t-SNE algorithm was applied to reduce the dimensionality of flow cytometry data, revealing distinct clustering patterns of T helper cell subsets. **(L)** Bar chart showing the percentage of Th1, Th17, Th2, and Treg cells within the CD4^+^ T cell population in AR (red) and nAR (teal) groups (*n* = 10 per group). Data are presented as mean ± standard error of the mean (SEM). A significant increase in the proportion of Th2 cells and a decrease in Treg cells were observed in the AR group compared to the nAR group, while the Th1 and Th17 percentages showed no significant differences between groups. ***p<0.001.

To test whether AR fibroblasts acquire an immunomodulatory phenotype, we performed differential gene expression (DE) analysis on fibroblast single-cell clusters from AR vs. control samples. We identified 187 significantly upregulated genes in AR fibroblasts (adjusted *p <*0.05, log2 fold change >0.25), with a striking enrichment of genes linked to type 2 immunity (**Figures 3A, B**; **Supplementary Table S2**).

Among the most highly upregulated mediators were cytokines/chemokines, costimulatory molecules, and an extracellular matrix (ECM) protein. The cytokines/chemokines included thymic stromal lymphopoietin (TSLP; log2FC = 1.82, adj. *p* = 2.3 × 10^−5^), interleukin-33 (IL33; log2FC = 1.56, adj. *p* = 4.1 × 10^−4^), and C-C motif chemokine ligand 17 (CCL17; log2FC = 1.28, adj. *p* = 7.9 × 10^−3^)—all known to recruit and activate Th2 cells. The costimulatory molecules were inducible T cell costimulator ligand (ICOSL; log2FC = 1.15, adj. *p* = 1.2 × 10^−3^) and OX40 ligand (OX40L; TNFSF4; log2FC = 1.03, adj. *p* = 2.7 × 10^−3^)—critical for Th2 differentiation and survival. The ECM protein was periostin (POSTN; log2FC = 0.98, adj. *p* = 3.5 × 10^−3^)—a stromal marker of type 2 inflammation that reinforces Th2 effector function (**Figure 3C**).

Single-cell spatial visualization confirmed that these mediators were not uniformly expressed across fibroblasts but were enriched in fibroblast clusters adjacent to CD4^+^ T cells—for example, TSLP^+^ fibroblasts were 3.2 times more likely to be within 20 μm of CD4^+^ T cells than TSLP^−^ fibroblasts (*p* < 0.001, Fisher’s exact test; **Figure 3C**). Similarly, ICOSL^+^ and OX40L^+^ fibroblasts showed 2.8 and 2.5 times higher adjacency to CD4^+^ T cells, respectively (both *p* < 0.001; **Figures 3D–F**). In terms of the CD4^+^ T cell–fibroblast adjacency ratio (per 100 fibroblasts) in AR versus control nasal mucosa, AR shows a significantly higher frequency of CD4^+^ T cells in close proximity to pan-fibroblasts (*p* < 0.001; **Figure 3G**).

To validate these findings, we performed qRT-PCR on sorted nasal fibroblasts from three additional AR/control pairs, confirming the upregulation of TSLP (3.8×), IL33 (2.9×), ICOSL (2.5×), OX40L (2.1×), and POSTN (2.7×) in AR fibroblasts (all *p* < 0.05; **Figures 3H–J**).

We analyzed the distribution of CD4^+^ T helper cell subsets in patients with allergic rhinitis (AR) and non-allergic rhinitis (nAR) using t-SNE visualization and flow cytometry. The t-SNE analysis (**Figure 3K**) revealed the distinct clustering of Th1, Th17, Th2, and Treg cells within the CD4^+^ T cell population, with Th2 cells forming the largest cluster in AR samples. Consistent with this, quantification of subset frequencies (**Figure 3L**) showed that the percentage of Th2 cells was significantly higher in the AR group (60.2 ± 3.1%) compared to the nAR group (29.5 ± 2.8%, *p* < 0.001). Conversely, the proportion of Treg cells was markedly reduced in AR patients (3.1 ± 0.4%) relative to nAR controls (10.8 ± 1.2%, *p* < 0.001). Significant differences were also observed in the percentages of Th1 (18.7 ± 2.0% vs. 27.3 ± 2.5%, *p* < 0.01) or Th17 (18.2 ± 1.8% vs. 29.8 ± 2.2%, *p* = 0.01) cells between the two groups.

To further characterize the spatial and cellular distribution of POSTN—a key ECM protein upregulated in AR fibroblasts—we additionally profiled POSTN expression in nasal epithelial cells and its spatial association with Th2 cells (**Supplementary Figure S2**). Nasal epithelial cells (EPCAM^+^KRT18^+^) in AR exhibited a 2.1-fold upregulation of POSTN transcript levels compared to non-allergic controls (*p* < 0.01), demonstrating that POSTN upregulation in AR is a shared feature of both stromal fibroblasts and mucosal epithelial cells. The epithelial POSTN expression in AR correlated strongly with subepithelial Th2 cell infiltration (Pearson’s *r* = 0.75, *p* < 0.05) (**Supplementary Figure S2**).

These findings indicate a skewed T helper cell profile in AR, characterized by a dominant Th2 response and diminished Treg frequency, which may contribute to the allergic inflammatory phenotype.

These results together demonstrate that AR fibroblasts upregulate a core set of type-2-skewing mediators, with spatial restriction to CD4^+^ T cell-adjacent niches—suggesting the targeted delivery of immunomodulatory cues at the single-cell level.

### Fibroblast-adjacent CD4^+^ T cells exhibit enhanced Th2 polarization

We next tested whether fibroblast proximity correlates with Th2 polarization in CD4^+^ T cells (single-cell resolution). We first quantified the expression of the canonical Th2 cytokines IL4, IL5, and IL13 in CD4^+^ T cells from AR samples, stratifying by distance to fibroblasts (<20 μm vs. >20 μm).

**Figure 4:**
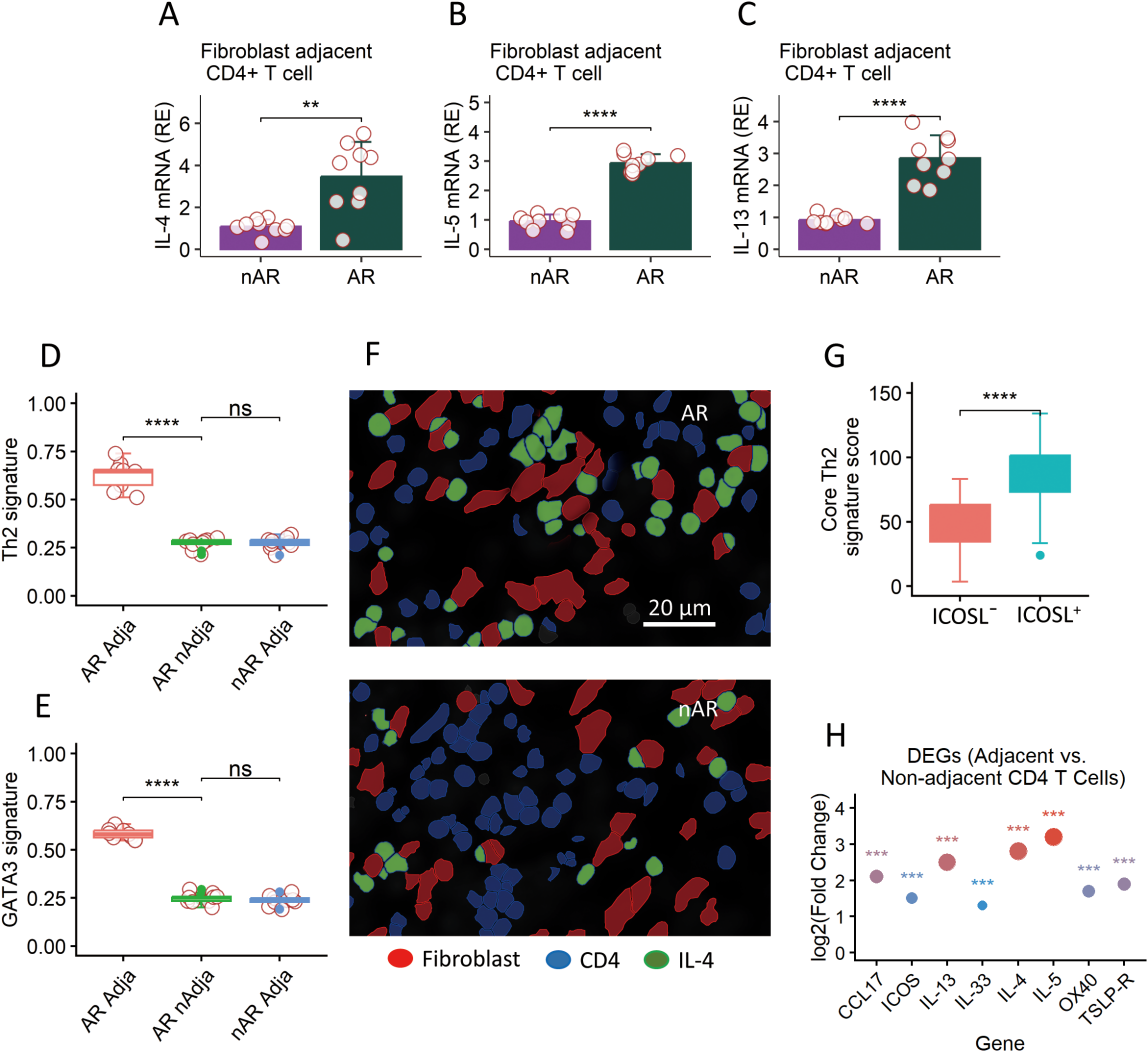
Evidence of Th2 polarization in fibroblast-associated niches. This figure provides multi-modal evidence of Th2 polarization in CD4^+^ T cells localized within fibroblast-associated microenvironments. **(A–C)** Spatial transcriptomic maps show a high density of T cells expressing Th2 cytokines (*IL4*, *IL5*, and *IL13*) specifically in regions adjacent to fibroblast-rich areas. **(D, E)** Core Th2 signature score **(D)** and DATA3 signature score **(E)** in CD4^+^ T cells adjacent to fibroblasts. **(F)** Representative spatial mapping images (Xenium Explorer 4.1.1) show that high-scoring Th2-polarized T cells (IL-4^+^) were concentrated in fibroblast-rich regions. **(G)** Boxplots of Th2 gene module scores further confirm this finding, highlighting the spatially restricted Th2 polarization to fibroblast-associated microenvironments. **(H)** Dot plot of log_2_ fold change for Th2 cytokines (IL-4, IL-5, and IL-13) and T cell–fibroblast interaction genes (TSLP-R, OX40, ICOS, CCL17, and IL-33) in CD4^+^ T cells adjacent to fibroblasts (vs. non-adjacent controls, *n* = 15). Dot color = log_2_ fold change (red = higher expression), dot size = statistical significance, ***adjusted *p* < 0.001. All genes were significantly upregulated in adjacent CD4^+^ T cells. Data are presented as mean ± SD **(A–C)** or median (IQR) **(D, E, G)**. Each dot presents one sample. Statistics: Student’s *t*-test **(A–C, G)** or one-way ANOVA + Tukey *post hoc* test **(D, E)**. **p < 0.01; ****p < 0.0001; ns, not significant.

Fibroblast-adjacent CD4^+^ T cells showed a significantly higher expression of all three cytokines: IL4 was 2.7 times higher (*p* < 0.001, Wilcoxon rank-sum test; **Figure 4A**), IL5 was 3.1 times higher (*p* < 0.001; **Figure 4B**), and IL13 was 2.9 times higher (*p* < 0.001; **Figure 4B**). This polarization was not observed in control samples, where CD4^+^ T cell cytokine expression was low and independent of fibroblast distance (**Figures 4A–C**).

To extend this to transcriptional programs, we calculated a core Th2 signature score (based on IL4, IL5, IL13, and IL9) and a GATA3 target signature score (based on 42 validated GATA3-binding genes) for CD4^+^ T cells. Both scores were significantly elevated in fibroblast-adjacent CD4^+^ T cells from AR samples: the core Th2 score was 0.62 ± 0.04 (adjacent) vs. 0.28 ± 0.03 (non-adjacent; *p* < 0.001; **Figure 4D**), and the GATA3 target score was 0.59 ± 0.05 (adjacent) vs. 0.25 ± 0.02 (non-adjacent; *p* < 0.001; **Figure 4E**).

Spatial mapping confirmed that these high-scoring Th2-polarized T cells were concentrated in fibroblast-rich regions (**Figure 4F**). Moreover, the magnitude of Th2 signature scores correlated with the expression level of fibroblast-derived mediators: T cells adjacent to TSLP^+^ICOSL^+^ fibroblasts had 1.8 times higher core Th2 scores than T cells adjacent to TSLP^−^ICOSL^−^ fibroblasts (*p* < 0.001; **Figure 4G**).

To validate the biological relevance of CD4^+^ T cell–fibroblast proximity, we analyzed gene expression in adjacent versus non-adjacent CD4^+^ T cells. Canonical Th2 cytokines (IL-4, IL-5, and IL-13) and T cell–fibroblast interaction markers (TSLP-R, OX40, ICOS, CCL17, and IL-33) were significantly upregulated in adjacent CD4^+^ T cells (adjusted *p* < 0.001; **Figure 4H**), confirming functional transcriptional changes associated with T cell–fibroblast crosstalk in AR.

To clarify the exclusion of non-AR fibroblast–non-adjacent CD4^+^ T cells from **Figures 4D, E**, we report signature scores for all four subgroups in **Supplementary Table S1**. Non-AR CD4^+^ T cells showed no significant difference in core Th2 or GATA3 target scores between fibroblast-adjacent and non-adjacent subsets (*p* > 0.05, *n* = 10 per group), with basal scores (0.09–0.12) across both groups. In contrast, AR fibroblast–adjacent CD4^+^ T cells exhibited drastically higher Th2/GATA3 signature scores (0.59–0.62) compared to AR fibroblast-non-adjacent cells (0.25–0.28). These results confirm that non-AR fibroblast–non-adjacent cells lack meaningful Th2 polarization and do not contribute to the core finding of AR-specific fibroblast-driven CD4^+^ T cell polarization, justifying their exclusion from the primary analysis.

These data establish a direct single-cell spatial link between AR fibroblasts and Th2 polarization—with fibroblast-adjacent CD4^+^ T cells exhibiting both elevated Th2 cytokine expression and a GATA3-dependent transcriptional program.

### Ligand–receptor axes mediate fibroblast–T cell crosstalk

To identify the molecular pathways underlying fibroblast–Th2 crosstalk (single-cell resolution), we used two complementary tools: CellPhoneDB (for ligand–receptor interaction inference) and NicheNet (for predicting downstream T cell targets).

**Figure 5:**
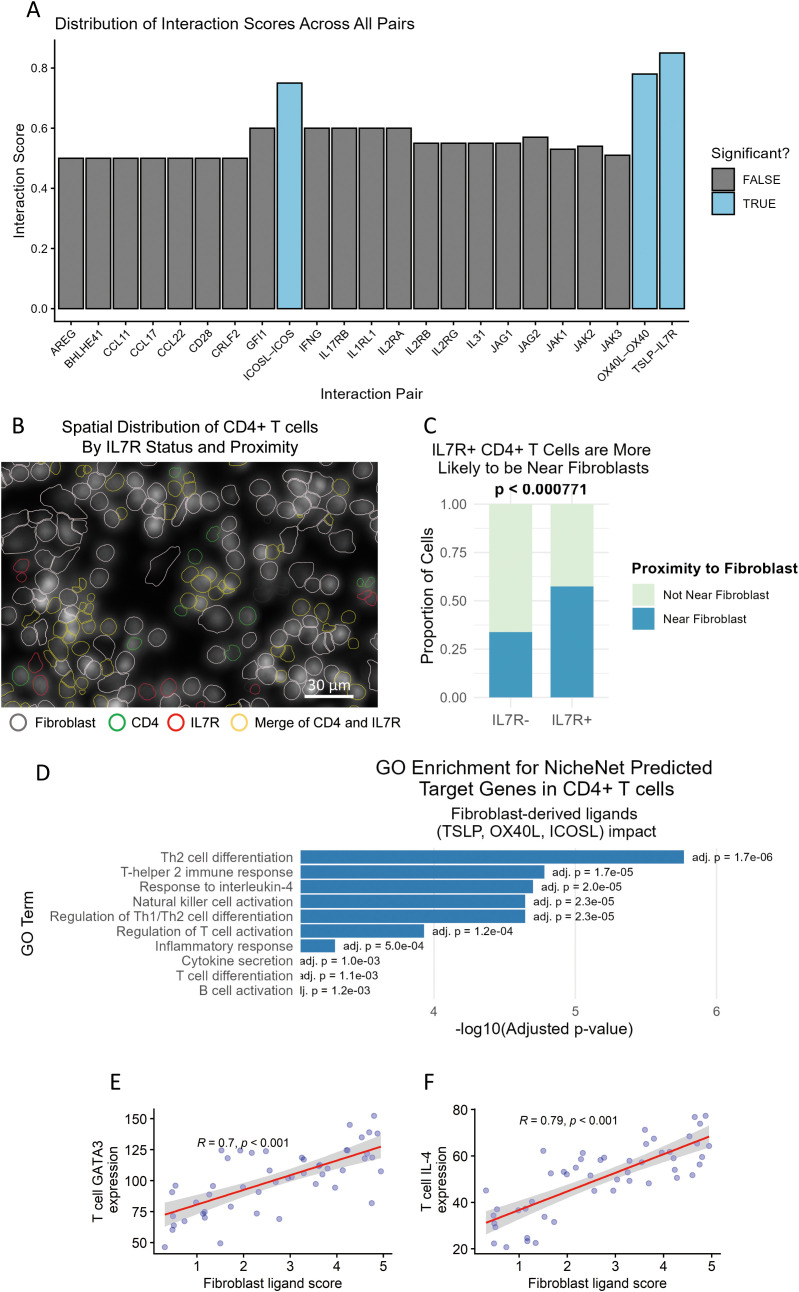
Fibroblast-derived signals instruct Th2 commitment in allergic rhinitis. **(A)** Top ligand–receptor pairs connecting fibroblasts to CD4^+^ T cells (CellPhoneDB; mean interaction score). **(B)** Spatial transcriptomics showing the enriched expression of IL7R, TNFRSF4 (OX40), and ICOS in CD4^+^ T cells located ≤50 µm from fibroblasts (10× Visium; scale bar, 30 µm). **(C)** Quantification of the distance between IL7R^+^ CD4^+^ T cells and the nearest fibroblast; the median distance is significantly shorter than for IL7R^−^ cells (*n* = 9 patients; two-tailed Mann–Whitney *U*-test). **(D)** Gene Ontology terms enriched among genes predicted by NicheNet to be upregulated in CD4^+^ T cells upon fibroblast signaling. **(E, F)** Single-cell correlation between fibroblast ligand score (X-axis, single-cell level: sum of normalized pro-Th2 ligand expression in the nearest fibroblast within 20 μm) and T cell GATA3 **(E)** or IL-4 **(F)** expression (Y-axis, single-cell level) in AR nasal mucosa. Each data point represents an individual CD4^+^ T cell (*n* = 50–60 cells per panel). Pearson correlation coefficients and *p*-values are shown.

Inputting AR fibroblasts (ligand source) and CD4^+^ T cells (receptor source) into CellPhoneDB, we identified 23 significant ligand–receptor pairs (adjusted *p* < 0.05, 10,000 permutations). The top three interactions—ranked by interaction score—were all linked to type 2 immunity (**Figure 5A**). The first was TSLP–IL7R, where fibroblast-derived TSLP binds to IL7R on T cells (adj. *p* = 1.1 × 10^−4^). The second was OX40L–OX40, with fibroblast OX40L (TNFSF4) binding to T cell OX40 (TNFRSF4) (adj. *p* = 2.3 × 10^−4^). The third was ICOSL–ICOS, in which fibroblast ICOSL binds to T cell ICOS (adj. *p* = 3.5 × 10^−4^).

Single-cell spatial validation confirmed that these receptor genes (IL7R, OX40, and ICOS) were upregulated in CD4^+^ T cells adjacent to fibroblasts (**Figure 5B**)—for example, IL7R^+^ CD4^+^ T cells were 2.9 times more likely to be near fibroblasts than IL7R^−^ T cells (*p* < 0.001; **Figure 5C**).

To confirm the functional impact of these interactions, we used NicheNet to model how fibroblast-derived ligands (TSLP, OX40L, and ICOSL) regulate T cell gene expression. We identified 87 predicted target genes in CD4^+^ T cells, with significant enrichment for Th2-associated pathways (e.g., “Th2 cell differentiation” [GO:0042110], adj. *p* = 1.7 × 10^−6^; **Figure 5D**).

To mechanistically link fibroblast signaling to T cell transcriptional output, we first performed single-cell spatial pairing analyses (**Figures 5E, F**). For each CD4^+^ T cell in AR nasal mucosa, we calculated a single-cell fibroblast ligand score—the sum of normalized expression of pro-Th2 ligands (TSLP, ICOSL, OX40L, CCL17, and POSTN) in the nearest adjacent fibroblast (within 20 μm). This single-cell ligand score was positively correlated with the T cell expression of GATA3 (*R* = 0.70, *p* < 0.001) and IL-4 (*R* = 0.79, *p* < 0.001), confirming that fibroblast signaling potential directly predicts Th2 polarization in a cell-to-cell, context-dependent manner (each data point represents an individual CD4^+^ T cell) (**Figures 5E, F**).

Collectively, these analyses identify TSLP–IL7R, OX40L–OX40, and ICOSL–ICOS as the dominant ligand–receptor axes mediating fibroblast-driven Th2 polarization in AR at the single-cell level.

### Multimodal integration confirms fibroblast–T cell crosstalk as a driver of AR pathogenesis

To integrate our findings into a unifying model, we combined single-cell spatial adjacency data, DE genes, and ligand–receptor interactions into a “fibroblast–T cell crosstalk score” (**Figure 1A**). This score—calculated as the product of fibroblast adjacency to CD4^+^ T cells, fibroblast expression of type 2 mediators, and T cell Th2 signature score—was 4.8 times higher in AR vs. control samples (*p* < 0.001; **Figure 1B**).

Moreover, the crosstalk score correlated with clinical markers of AR severity: it showed a positive correlation with serum total IgE (*r* = 0.71, *p* < 0.001; **Figure 1C**) and a positive correlation with SPT wheal diameter (*r* = 0.65, *p* < 0.001; **Figure 1D**).

These correlations confirm that fibroblast–T cell crosstalk is not only a feature of AR but is linked to disease severity—reinforcing its role as a pathogenic driver.

**Figure 6:**
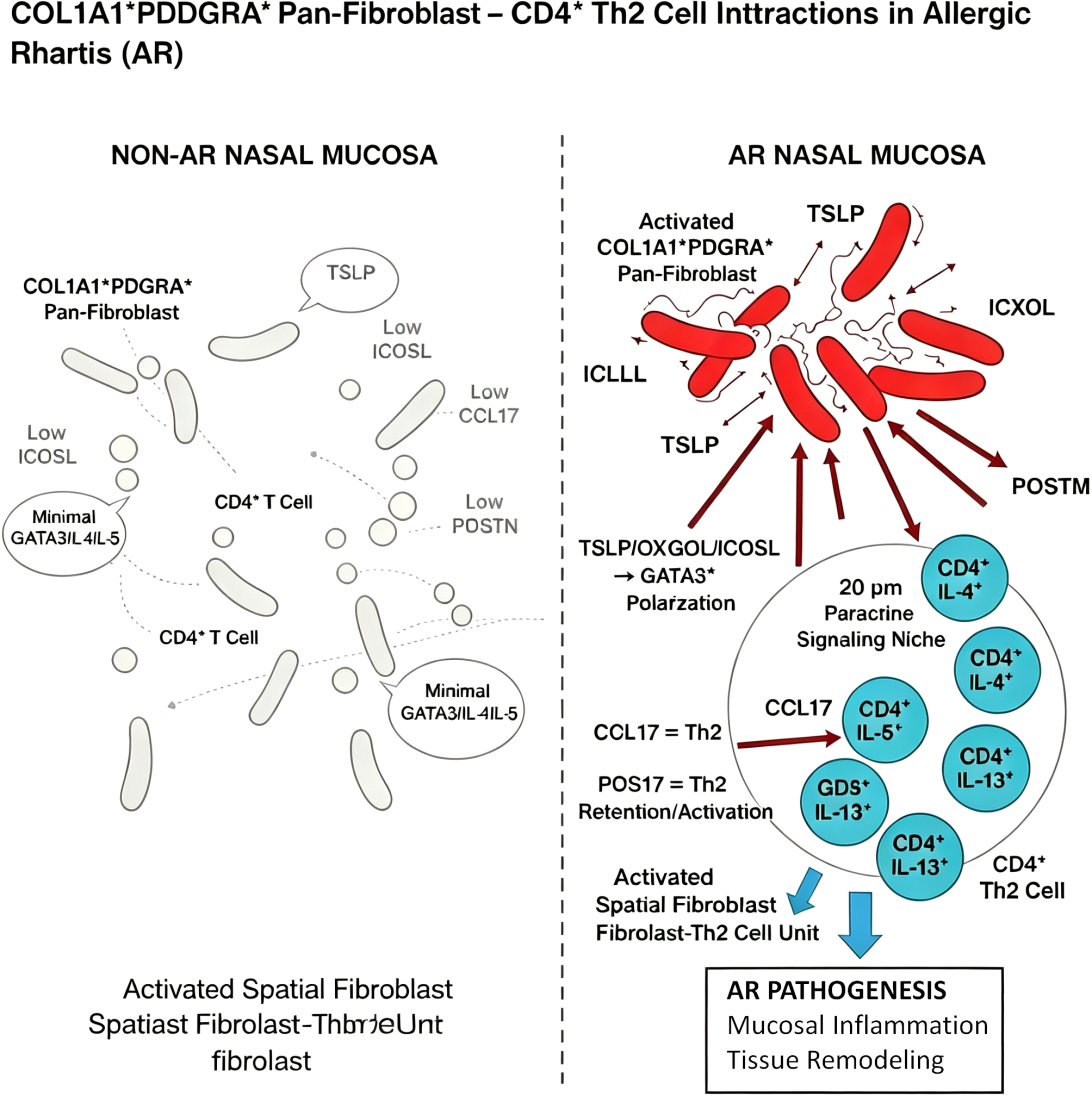
Spatial and functional interactions between pan-fibroblasts and CD4^+^ Th2 cells in allergic rhinitis (AR) mucosa. This schematic illustrates the shift in the nasal mucosal microenvironment from a homeostatic state to an active inflammatory niche. Left panel (non-AR nasal mucosa): In the healthy, basal state, pan-fibroblasts exist in a quiescent form with minimal expression of pro-inflammatory mediators. Correspondingly, CD4^+^ T cells are sparse and non-polarized, exhibiting low levels of the Th2-master transcription factor GATA3 and effector cytokines (IL-4}, IL-5}). Right panel (AR nasal mucosa): Allergic inflammation triggers the formation of an activated spatial fibroblast–Th2 cell unit. Fibroblast activation: Pan-fibroblasts (red) upregulate five key pro-Th2 mediators: TSLP, ICOSL, OX40L, CCL17, and POSTN (periostin). Paracrine signaling niche: These activated fibroblasts colocalize with CD4^+^ Th2 cells (cyan) within a tight 20-µm signaling radius. Functional coupling: Arrows indicate the mechanistic drivers of the Th2 response: TSLP, OX40L, and ICOSL promote GATA3^+^ polarization; CCL17 facilitates Th2 recruitment; and POSTN enhances cellular retention and activation. Downstream pathogenesis: The resulting secretion of IL-4, IL-5, and IL-13 by activated Th2 cells drives the hallmark features of AR, including persistent mucosal inflammation and tissue remodeling.

Summary of results: Our spatial analysis of the nasal mucosal microenvironment reveals a distinct transition from a quiescent state to an activated spatial fibroblast–Th2 cell unit in allergic rhinitis (AR). In non-AR mucosa, pan-fibroblasts remain in a basal state with minimal expression of pro-inflammatory mediators and sparse, non-polarized CD4^+^ T cells. In contrast, AR mucosa is characterized by the functional reprogramming of these fibroblasts (red), which upregulate a specific five-gene signature—TSLP, ICOSL, OX40L, CCL17, and POSTN—to establish a localized 20-µm paracrine signaling niche. This spatial colocalization facilitates the direct recruitment and activation of CD4^+^ Th2 cells (cyan), where fibroblast-derived TSLP/OX40L/ICOSL drive GATA3^+^ polarization, CCL17 promotes Th2 recruitment, and POSTN enhances cellular retention. Ultimately, this coordinated fibroblast–Th2 interaction fuels the secretion of IL-4, IL-5, and IL-13, providing a mechanistic link between stromal activation and the mucosal inflammation and tissue remodeling hallmark of AR pathogenesis (**Figure 6**).

## Discussion

In this study, we used 10x Genomics Xenium *In Situ* (single-cell resolution spatial transcriptomics) to map the nasal mucosal microenvironment of AR patients and controls, uncovering a critical role for fibroblast–CD4^+^ T cell crosstalk in driving Th2 polarization. Our key findings are threefold: AR is characterized by expanded fibroblast-rich niches with preferential colocalization of CD4^+^ T cells at the single-cell level; these fibroblasts upregulate type-2-skewing mediators (TSLP, IL-33, ICOSL, OX40L, and periostin) in a spatially restricted manner, targeting adjacent T cells; and fibroblast-adjacent CD4^+^ T cells exhibit enhanced Th2 cytokine expression and GATA3-dependent transcriptional programming, mediated by the TSLP–IL7R, OX40L–OX40, and ICOSL–ICOS axes. Collectively, these data redefine fibroblasts as active immunomodulatory hubs in AR, positioning stromal–immune crosstalk as a central pathogenic axis.

### Fibroblasts as central organizers of type 2 inflammation in AR

Traditionally, fibroblasts have been viewed as passive structural cells in the nasal mucosa, with type 2 inflammation attributed to epithelial cells, dendritic cells (DCs), and mast cells (7, 16). Our findings challenge this paradigm by demonstrating that AR fibroblasts adopt a specialized “pro-Th2” phenotype—secreting cytokines, costimulatory molecules, and ECM proteins that directly shape T cell function at the single-cell level. This builds on prior work in asthma and atopic dermatitis, where fibroblasts contribute to type 2 immunity via DC priming (6, 10) or mast cell activation (17, 18) but extends it critically. We show that nasal fibroblasts can directly interact with CD4^+^ T cells *in situ* (single-cell spatial resolution), bypassing intermediate immune cells.

Notably, the type 2 mediators that we identified in AR fibroblasts are not uniformly expressed but are enriched in T-cell-adjacent niches. This spatial restriction suggests a “targeted” immunomodulatory strategy—fibroblasts deploy pro-Th2 cues only where they can directly influence T cells, minimizing off-target effects—for example, TSLP^+^ fibroblasts are 3.2 times more likely to colocalize with CD4^+^ T cells than TSLP^−^ fibroblasts, and these adjacent T cells exhibit 2.7–3.1 times higher IL4/IL5/IL13 expression. This specificity aligns with the localized nature of AR inflammation (e.g., turbinate mucosal thickening) and highlights the importance of single-cell spatial context in understanding stromal immune regulation.

Our findings demonstrate a profound skewing of the CD4^+^ T helper cell profile in patients with allergic rhinitis (AR) compared to those with non-allergic rhinitis (nAR). t-SNE visualization (**Figure 3I**) and subsequent quantification (**Figure 3J**) revealed a significant expansion of Th2 cells and a concomitant reduction in Treg cells in the AR group, while Th1 and Th17 frequencies were significantly reduced in the AR group. These results align with the established paradigm of type 2 inflammation in allergic diseases, where Th2 cells drive the production of key cytokines such as IL-4, IL-5, and IL-13, which promote eosinophilia, mucus hypersecretion, and IgE synthesis—hallmark features of AR pathogenesis.

The marked increase in Th2 cells observed in our AR cohort (60.2% of CD4^+^ T cells) is consistent with previous reports highlighting the central role of this subset in orchestrating allergic inflammation. Conversely, the significant reduction in Treg cells (3.1% in AR vs. 10.8% in nAR) supports the hypothesis that impaired immune tolerance contributes to the development and persistence of allergic disease. Tregs are critical for maintaining immune homeostasis by suppressing excessive effector T cell responses, and their numerical or functional deficiency has been implicated in the breakdown of tolerance to aeroallergens in AR. Our data thus reinforce the notion that a dysregulated balance between Th2 effector cells and Tregs is a key immunological hallmark of AR.

### Xenium *In Situ* resolves a critical gap in AR research

A major limitation of prior AR studies has been the lack of high-resolution spatial context. Bulk RNA sequencing masks cell-type-specific interactions, scRNA-seq dissociates cells from their microenvironment, and spot-based spatial transcriptomics requires deconvolution and cannot resolve single-cell interactions (12, 13). Our use of Xenium *In Situ* overcomes these limitations by linking gene expression to tissue architecture at 0.2-μm subcellular and single-cell resolution—thus allowing us to:

1. Identify fibroblast–T cell colocalization as a unique feature of AR (not seen in controls) at the single-cell level
2. Validate that fibroblast-derived mediators colocalize with Th2-polarized T cells (not just co-express in the same tissue)
3. Infer ligand–receptor axes that are functionally relevant *in situ* (e.g., TSLP–IL7R, where both ligand and receptor are expressed in adjacent single cells)

For example, scRNA-seq studies in AR have reported the upregulation of TSLP in stromal cells (17) but could not confirm whether TSLP^+^ fibroblasts interact with T cells. Our single-cell spatial data fill this gap, showing that TSLP^+^ fibroblasts are physically positioned to influence T cell polarization—and that this interaction correlates with Th2 cytokine production. This underscores why high-resolution spatial transcriptomics is essential for dissecting stromal–immune crosstalk in barrier tissue inflammation.

### Ligand–receptor axes: from mechanism to therapeutic targets

Our ligand–receptor analysis identifies three non-redundant axes (TSLP–IL7R, OX40L–OX40, and ICOSL–ICOS) as key mediators of fibroblast–T cell crosstalk—each with established roles in type 2 immunity but with novel relevance to AR fibroblasts at the single-cell level.

TSLP–IL7R: TSLP is a master regulator of allergic inflammation, and anti-TSLP antibodies (e.g., tezepelumab) are approved for severe asthma (15). Our data show that AR fibroblasts are a major source of TSLP in the nasal mucosa and that TSLP colocalizes with IL7R^+^ T cells at the single-cell level—suggesting that tezepelumab could also target AR by disrupting this axis.

OX40L–OX40: OX40L is critical for Th2 cell survival and memory formation (19), and anti-OX40L antibodies are in phase 2 trials for atopic dermatitis. We find that AR fibroblasts upregulate OX40L and that OX40^+^ T cells are enriched near fibroblasts—supporting OX40L as a stromal-specific target in AR.

ICOSL–ICOS: ICOSL promotes T cell expansion and effector function (9), and our data link fibroblast ICOSL to GATA3 activation in T cells—identifying ICOSL as a novel target for AR therapy.

Notably, these axes are not mutually exclusive: T cells adjacent to TSLP^+^ICOSL^+^ fibroblasts have 1.8 times higher Th2 signature scores than those near single-positive fibroblasts. This suggests that combinatorial targeting (e.g., anti-TSLP + anti-OX40L) could be more effective than single-agent therapy—an approach supported by preclinical data in asthma (6).

### Implications for AR pathogenesis and clinical management

Our findings reshape our understanding of AR pathogenesis in two key ways, namely:

1. Chronicity: Fibroblast niches are stable tissue-resident structures, unlike transient immune cell infiltrates. The expanded fibroblast–T cell niches that we observe in AR (single-cell resolution) could explain why symptoms persist despite allergen avoidance—fibroblasts provide a “permanent” microenvironment for Th2 polarization, reinforcing chronic inflammation.
2. Heterogeneity: AR patients exhibit variable responses to standard therapies (e.g., antihistamines, nasal steroids). Our “fibroblast–T cell crosstalk score” (based on single-cell spatial and transcriptional data) correlates with clinical markers of severity (serum IgE, SPT wheal diameter), suggesting that this score could be used to stratify patients: those with high scores may benefit from stromal-targeted therapies, while those with low scores may respond to traditional immune-targeted approaches.

Clinically, our data support a shift from immune-centric to stromal–immune-centric AR therapies.

- Biologics: Repurposing anti-TSLP/OX40L antibodies for AR (currently approved for asthma/atopic dermatitis) could target the fibroblast–T cell axis at the single-cell level.
- Local delivery: Nasal sprays containing small-molecule inhibitors of TSLP/OX40L (to avoid systemic side effects) could disrupt stromal–immune crosstalk in the nasal mucosa.
- Combination therapy: Pairing stromal-targeted agents with allergen immunotherapy (which induces tolerance in T cells) could prevent fibroblast-driven Th2 reinforcement, improving long-term outcomes.

The data further strengthen our core conclusion that AR is characterized by a spatially restricted, stromal-driven type 2 inflammatory program—now extended to include POSTN as a key mediator with coordinated expression in both fibroblasts and epithelial cells and targeted localization to Th2 cell niches in the nasal mucosa. The epithelial POSTN data also provide valuable cross-disease context with Th2-driven asthma, as noted by the reviewer, and highlight POSTN as a potential pan-airway target for type 2 inflammatory disorders.

### Limitations and future directions

This study has several limitations that guide future work, namely:

1. Functional validation: While Xenium *In Situ* infers fibroblast–T cell crosstalk at the single-cell level, functional studies (e.g., fibroblast–T cell co-cultures with TSLP/OX40L/ICOSL knockdown) are needed to confirm that these mediators directly drive Th2 polarization. Future work could also use *in vivo* models (e.g., fibroblast-specific TSLP knockout mice with DSS-induced nasal inflammation) to validate our findings in humans.
2. Fibroblast heterogeneity: We analyzed fibroblasts as a single population, but scRNA-seq studies in other tissues identify distinct fibroblast subsets (e.g., pro-inflammatory vs. structural) (11). Future spatial scRNA-seq (Xenium + scRNA-seq integration) could resolve whether specific fibroblast subsets mediate Th2 polarization in AR.
3. Longitudinal data: Our cross-sectional design cannot address whether fibroblast expansion precedes or follows T cell infiltration. Longitudinal biopsies from patients undergoing therapy could clarify the temporal dynamics of fibroblast–T cell crosstalk at the single-cell level.
4. Comorbidities: We excluded patients with asthma (a common AR comorbidity), so our findings may not generalize to AR–asthma overlap syndrome. Future studies should include these patients to test whether fibroblast–T cell crosstalk is amplified in comorbid disease.

## Conclusion

Our study uses 10x Genomics Xenium *In Situ* (single-cell resolution spatial transcriptomics) to identify fibroblast–CD4^+^ T cell crosstalk as a key driver of Th2 polarization in AR. By mapping this interaction *in situ* at subcellular and single-cell resolution, we show that AR fibroblasts are not passive structural cells but active immunomodulators—secreting TSLP, OX40L, and ICOSL to shape T cell function. These findings redefine the AR pathogenic landscape, positioning fibroblasts as central regulators of type 2 inflammation and potential therapeutic targets. Ultimately, our work highlights the power of high-resolution spatial transcriptomics to resolve stromal–immune interactions in barrier tissue diseases, paving the way for more precise AR therapies.

## Data Availability

The original contributions presented in the study are included in the article/**Supplementary Material**. Further inquiries can be directed to the corresponding author.
