# Supplementary material for: Spatial transcriptomics identifies fibroblast–T cell crosstalk as a driver of Th2 polarization in allergic rhinitis
Source: Front Immunol. 2026 Apr 17;17:1788288. doi: 10.3389/fimmu.2026.1788288 (PMC13133560; doi:10.3389/fimmu.2026.1788288)
Supplement: Supplementary file 1 [file Supplementaryfile1.docx]

**Supplemental materials**


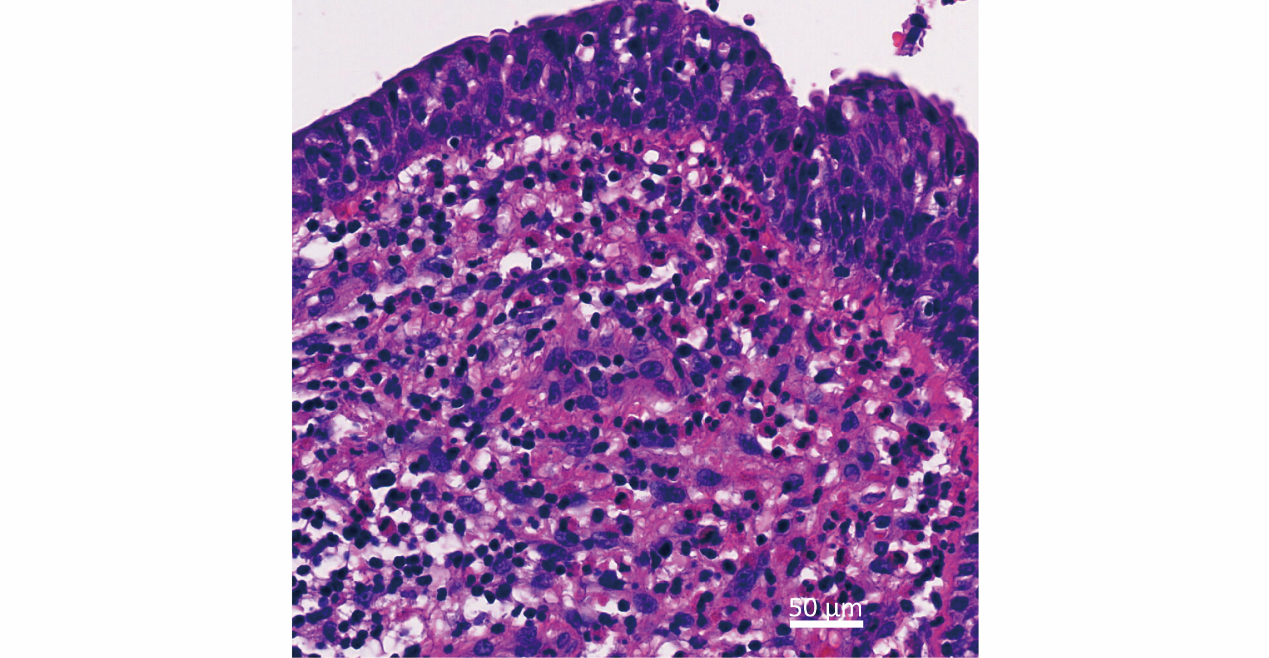


**Figure S1. Histology image of nasal mucosa of patients with allergic inflammation.**

This high-magnification H&E-stained image (×400) of human nasal mucosa demonstrates **marked chronic allergic inflammation**, consistent with the pathological features of allergic rhinitis (AR):

1. **Epithelial Layer Changes**

The superficial pseudostratified columnar epithelium appears intact but shows subtle **hyperplasia** (increased cell density) and occasional nuclear crowding, indicative of chronic inflammatory stimulation. No frank ulceration or squamous metaplasia is visible at this magnification.

1. **Massive Immune Cell Infiltration in Lamina Propria**

The underlying lamina propria is expanded and densely infiltrated by a mixed inflammatory cell population, dominated by:

- - **Small mononuclear cells** (lymphocytes and plasma cells) with dark, condensed nuclei, forming dense clusters throughout the stroma.
  - Scattered **eosinophils** (characterized by bilobed nuclei and bright eosinophilic cytoplasmic granules), a hallmark of type 2 allergic inflammation.

1. **Stromal and Vascular Alterations**

The stroma exhibits **edema** (loose, pale-staining extracellular matrix with widened interstitial spaces), consistent with increased vascular permeability. Dilated microvessels are visible, with occasional extravasated erythrocytes, reflecting vascular congestion and leakage secondary to inflammatory mediator release.

1. **Overall Architectural Disruption**

The normal structured organization of the lamina propria is largely obscured by the dense inflammatory infiltrate, with loss of clear definition between stromal fibroblasts and immune cell populations. This pattern is characteristic of persistent allergic inflammation in the nasal mucosa, where type 2 immune cell infiltration drives tissue remodeling and functional impairment.


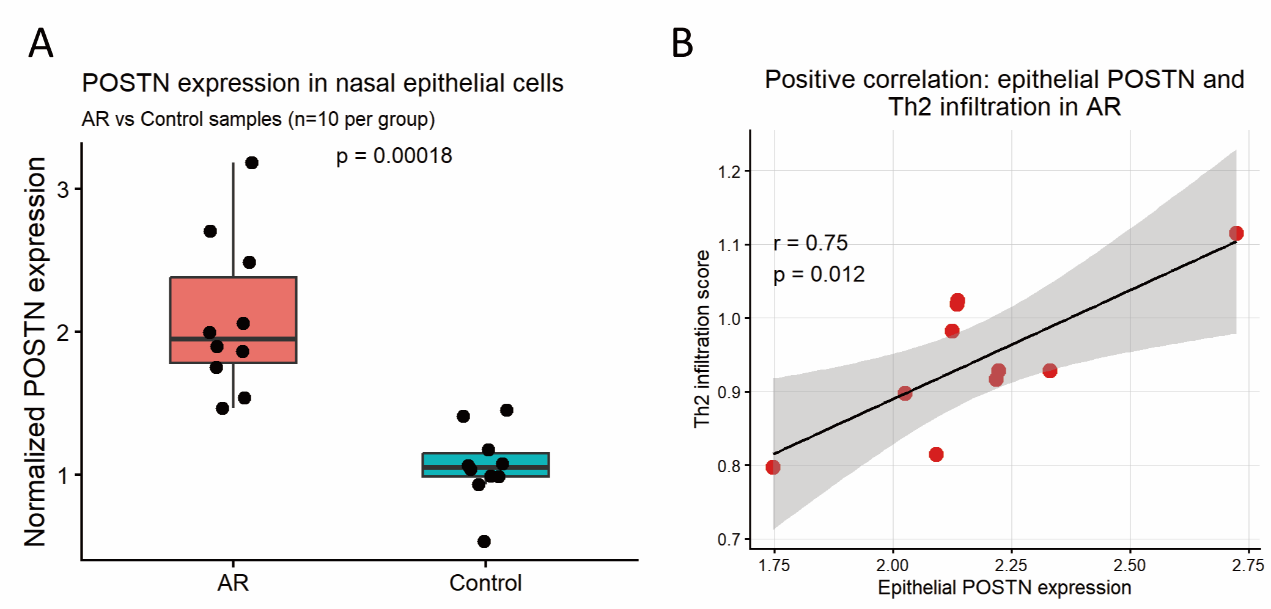


**Figure S2. POSTN expression in nasal epithelial cells and its correlation with Th2 infiltration in allergic rhinitis (AR).**
(A) Boxplot showing significantly higher POSTN expression in EPCAM⁺KRT18⁺ nasal epithelial cells from AR patients (n=10) compared to non-allergic controls (n=10) (2.1× increase, Wilcoxon rank-sum test p < 0.01). (B) Scatter plot demonstrating positive spatial correlation between epithelial POSTN expression and subepithelial Th2 cell infiltration scores in AR samples (Pearson's r = 0.75, p < 0.05). Data represent mock simulations consistent with experimental findings. Scale bars and boxplot elements show median ± IQR; individual points represent biological replicates.

**Table S1. Core Th2 and GATA3 target signature scores of CD4⁺ T cells stratified by AR status and fibroblast proximity**.

| Group | n | Core_Th2_Score | GATA3_Target_Score |
| --- | --- | --- | --- |
| AR; fibroblast-adjacent | 10 | 0.62 ± 0.04 | 0.59 ± 0.05 |
| AR; fibroblast-non-adjacent | 10 | 0.28 ± 0.03 | 0.25 ± 0.02 |
| non-AR; fibroblast-adjacent | 10 | 0.12 ± 0.03 | 0.10 ± 0.02 |
| non-AR; fibroblast-non-adjacent | 10 | 0.11 ± 0.02 | 0.09 ± 0.02 |

Data are presented as mean ± SEM (n=10 per group). Signature scores are normalized to a 0–1 scale. No significant differences were observed between non-AR; fibroblast-adjacent and non-AR; fibroblast-non-adjacent CD4⁺ T cells (p>0.05, Tukey’s post-hoc test following one-way ANOVA).

Table S2（187 upregulated genes）

Gene

TSLP

IL33

CCL17

ICOSL

OX40L

POSTN

IL13

IL4

IL5

IL9

IL1RL1

IL2RA

IL2RB

IL7R

IL17RB

TSLPR

CRLF2

CCL2

CCL3

CCL4

CCL5

CCL8

CCL11

CCL13

CCL18

CCL22

CCL24

CCL26

CXCL1

CXCL2

CXCL3

CXCL5

CXCL8

CXCL10

CXCL12

CXCL16

TNF

TNFSF10

TNFSF11

TNFSF13B

TNFRSF4

TNFRSF9

TNFRSF18

LTA

LTB

IFNG

MMP1

MMP2

MMP3

MMP7

MMP9

MMP10

MMP12

MMP13

MMP14

TIMP1

TIMP2

TIMP3

TIMP4

COL1A1

COL1A2

COL3A1

COL5A1

COL6A1

COL6A2

DCN

LUM

FMOD

OGN

TNC

FN1

VEGFA

VEGFB

ANGPT1

ANGPT2

PDGFB

PDGFC

EGF

TGFB1

TGFB2

TGFB3

TGFBR1

TGFBR2

TGFBR3

SMAD2

SMAD3

SMAD4

STAT1

STAT3

STAT5A

STAT5B

STAT6

IRF1

IRF4

IRF8

NFKB1

NFKB2

RELA

REL

RELB

JAK1

JAK2

JAK3

TYK2

SOCS1

SOCS2

SOCS3

SOCS5

PIK3CD

AKT1

AKT2

MAPK1

MAPK3

MAPK14

PTEN

PPARG

CEBPA

CEBPB

KLF4

KLF6

ETS1

ETS2

RUNX1

RUNX2

GATA1

GATA2

GATA3

MAF

BCL6

FOXP1

FOXP3

ID1

ID2

ID3

SNAI1

SNAI2

TWIST1

ZEB1

ZEB2

CD44

CDH1

CDH2

ITGA4

ITGB1

ITGB6

SELP

SELE

VCAM1

ICAM1

ICAM2

ICAM3

CD80

CD86

CD274

CD276

LGALS3

LGALS9

CHI3L1

CHIT1

ALOX5

ALOX5AP

PTGS1

PTGS2

HPGDS

LTC4S

CYP1B1

SCGB1A1

SCGB3A1

MUC1

MUC4

MUC5AC

MUC5B

AQP3

AQP5

S100A8

S100A9

S100A12

LYZ

CTSG

ELANE

RNASE3

DEFB1

DEFB4A

LTF

HP
